# High-Throughput Top-Down Fabrication of Uniform Magnetic Particles

**DOI:** 10.1371/journal.pone.0037440

**Published:** 2012-05-31

**Authors:** Julia Litvinov, Azeem Nasrullah, Timothy Sherlock, Yi-Ju Wang, Paul Ruchhoeft, Richard C. Willson

**Affiliations:** 1 Department of Biomedical Engineering, University of Houston, Houston, Texas, United States of America; 2 Department of Electrical and Computer Engineering, University of Houston, Houston, Texas, United States of America; 3 Department of Chemical and Biomolecular Engineering, University of Houston, Houston, Texas, United States of America; 4 The Methodist Hospital Research Institute, Houston, Texas, United States of America; Aristotle University of Thessaloniki, Greece

## Abstract

Ion Beam Aperture Array Lithography was applied to top-down fabrication of large dense (10^8^–10^9^ particles/cm^2^) arrays of uniform micron-scale particles at rates hundreds of times faster than electron beam lithography. In this process, a large array of helium ion beamlets is formed when a stencil mask containing an array of circular openings is illuminated by a broad beam of energetic (5–8 keV) ions, and is used to write arrays of specific repetitive patterns. A commercial 5-micrometer metal mesh was used as a stencil mask; the mesh size was adjusted by shrinking the stencil openings using conformal sputter-deposition of copper. Thermal evaporation from multiple sources was utilized to form magnetic particles of varied size and thickness, including alternating layers of gold and permalloy. Evaporation of permalloy layers in the presence of a magnetic field allowed creation of particles with uniform magnetic properties and pre-determined magnetization direction. The magnetic properties of the resulting particles were characterized by Vibrating Sample Magnetometry. Since the orientation of the particles on the substrate before release into suspension is known, the orientation-dependent magnetic properties of the particles could be determined.

## Introduction

Nano- and micro-particles, especially spheres and nanoshells, play a growing role in medical diagnostic and therapeutic technologies. Magnetic particles are of particular interest for sample preparation, cell purification, imaging, and the fabrication and functionalization of advanced materials [1]. Magnetic particles have been manufactured in many different ways, including chemical synthesis [2], hydrothermal treatment [3]_,_ photochemical metal deposition [4], and sol-gel fabrication [5], but challenges remain in engineering particles with uniform size and composition, and resistance to corrosion in biological solutions. Non-spherical particles are expected to give superior performance in many applications, including imaging and diagnostics, but access to uniform non-spherical particles is highly constrained by the limitations of available synthetic approaches [6], [7]. Independent control of size, shape, uniformity and composition is not possible during synthesis by conventional methods.

In this work, a unique Ion Beam Aperture Array Lithographic (AAL) nanoparticle fabrication technique was developed which allows inexpensive direct formation of dense arrays (up to 10^9^–10^10^ particles/cm^2^) of shaped magnetic particles with very high throughput (hundreds of cm^2^/hr; hundreds of times faster than electron beam lithography). The particles can be made from a large variety of materials, and multiple layers can be deposited to form e.g., magnetic-core gold particles.

In this work, AAL was used to pattern large arrays of periodic structures in resist as a template for forming suspendable particles with engineered shape and composition. The pattern consists of an array of circular or rectangular features used to form disk-shaped permalloy-core gold particles with 5, 3, and 1 micron, and 300 nm diameters, and rod-shaped 1.2×0.3 µm permalloy-core particles covered with a 10 nm gold shell, which allows facile surface modifications [8], [9], [10], [11], [12]. All magnetic particles were made using physical vapor deposition in the presence of a static, in-plane magnetic field of 40 Oersted applied to the surface of the samples. The thickness of the layer of deposited permalloy was varied, and VSM measurements were made to characterize the particles’ magnetic properties. Since the orientation of the particles on the substrate before release into suspension is known, the orientation-dependent magnetic properties of the particles can be studied. In contrast to most other particles [13], [8], [14], [15], [16], the present particles of interest are made with a magnetic core, which allows them to be manipulated in solution [17], [18], [19] and also to be magnetized in a needed direction during particle fabrication. The particles are made with a gold shell which facilitates surface modification [8], [9], [10], [11], [12].

Particles with magnetic properties have customarily been manufactured by solution synthesis approaches [16]. While these approaches are efficient, scalable, and well-established, it is not easy to align the magnetic orientation and adjust the remnant magnetization magnitude for non-symmetric particles. Also, it is difficult to characterize their anisotropic magnetic properties since the particles are typically in liquid suspension [20], and it is nearly impossible to orient the particles on a surface with their magnetization vectors aligned. Using the AAL technique, the particles are made with known pre-determined magnetic orientation, which allows characterization of their anisotropic magnetic properties using VSM.

## Materials and Methods

### Ion Beam Aperture Array Lithography Apparatus

Our ion beam aperture array lithography has been described previously [21], [22], [23]. In this process, as used here, a 1×1 cm^2^ mask containing an array of 10^6^ square openings of 5 µm on 10 µm pitch is illuminated by a broad (5 cm) beam of 5–8 keV He ions. Ion beamlets formed when the mask is illuminated by the beam are used to expose the resist with the array pattern. The mask is 1.2 meters from the ion source, and a proximity gap of 600 µm is maintained between the mask and the substrate. The substrate is moved behind the mask using a coarse mechanical stage for large movements and a fine-motion, flexure stage for small displacements. The resolution of the AAL tool is better than 50 nm and depends primarily on the quality of the mask and the proximity gap [24]. The size and uniformity of the fabricated particles depend on the size and uniformity of the mask openings, and on the time of exposure of the sample to the ion beam.

### Particle Formation and Release

**Figure 1:**
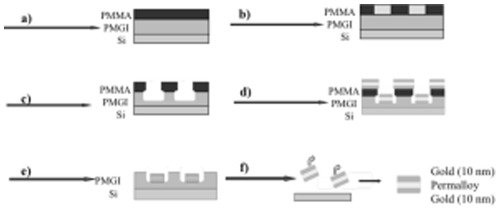
Particle Fabrication Sequence. Spin-coating of PMGI and PMMA on a clean silicon wafer (a) is followed by exposure of the sample to a broad beam of helium ions through a stencil mask to form the pattern (b). During development, the exposed areas of PMMA wash away, and a subsequent etch in TMAH removes the PMGI layer underneath the PMMA openings (c). The particles of interest are evaporated as stacked layers of 10 nm gold, 10 nm of permalloy, and 10 nm gold (d). A lift-off procedure removes the evaporated metal on top of the PMMA layer (e) and the PMGI layer is etched in TMAH solution (f) to release the particles.

Figure 1 outlines the major steps of particle fabrication. (a) A layer of 300 nm polymethylglutarimide (PMGI) is spin-coated on a silicon wafer as a sacrificial layer for later particle removal and baked at 180°C for 20 min. A layer of 50 nm of poly(methyl methacrylate) (PMMA) is spin-coated as beam resist and baked at 180°C for 1 hour. (b) The PMMA layer is exposed using Ion Beam Lithography through a 1 cm^2^ stencil mask that contains 5 µm openings on a 10 µm pitch. (c) The pattern is then developed in 3∶1 IPA/MIBK solution for 30 seconds and rinsed in IPA for 30 seconds before drying in a stream of dry nitrogen. This step washes away the area of the resist exposed by the ion beam and leaves the pattern of PMMA on the PMGI layer. The PMGI is partially etched in 2.3% TMAH solution to create an undercut and allow dissolution of the remaining PMGI layer during the particle lift-off step. (d) Particles are formed on the base of the opening by thermal evaporation of gold (gold wire, 0.1 mm diameter, Premion®, 99.998%) and NiFe (“permalloy” pellets, 79% Fe, 21% Ni, 3 mm diameter, Premion®, 99.99%). The metals were evaporated as sandwiches of 10 nm gold/permalloy of varied thickness/10 nm gold, as shown in Figure 1, to create layered magnetic particles surrounded by gold. (e) The PMMA was dissolved in an acetone bath to lift off the layer of metal that was evaporated on top of the PMMA, leaving behind an array of multilayered metal particles embedded in PMGI. (f) To release the particles, the sample was placed into fresh 2.3% TMAH in a 1.5 mL Eppendorf tube to dissolve the PMGI support. After rinsing with DI water, the particles were re-suspended in 10% citrate solution, or stored in the original 2.3% TMAH surface-release solution. Since the TMAH solution is very basic (pH 13.7), the particles are negatively charged and are electrostatically stabilized against aggregation; this is useful for long-term storage. The particles can easily be exchanged from TMAH into another buffer while immobilized by an external magnetic field.

### Magnetic Characterization

A Lakeshore Vibrating Sample Magnetometer (VSM) (Lakeshore, Inc., Westerville, OH) was used for magnetic characterization of the particle arrays as a function of applied magnetic field. An electromagnet and power supply are used to generate a constant magnetic field that is used to magnetize the sample. When the sample is vibrated the changes in magnetic flux produce a voltage that is detected by detection coils, which is proportional to the magnetic moment of the sample.

Since permalloy has a face-centered cubic (FCC) structure, it has only one easy axis of magnetization, along the (111) direction [25], [26]. The gold/permalloy/gold films of interest were grown in the presence of a magnetic field and the orientation of the easy-axis was chosen to be in-plane with the surface for all particles, in the (100) direction.

### Particle Fabrication and Characterization

**Figure 2:**
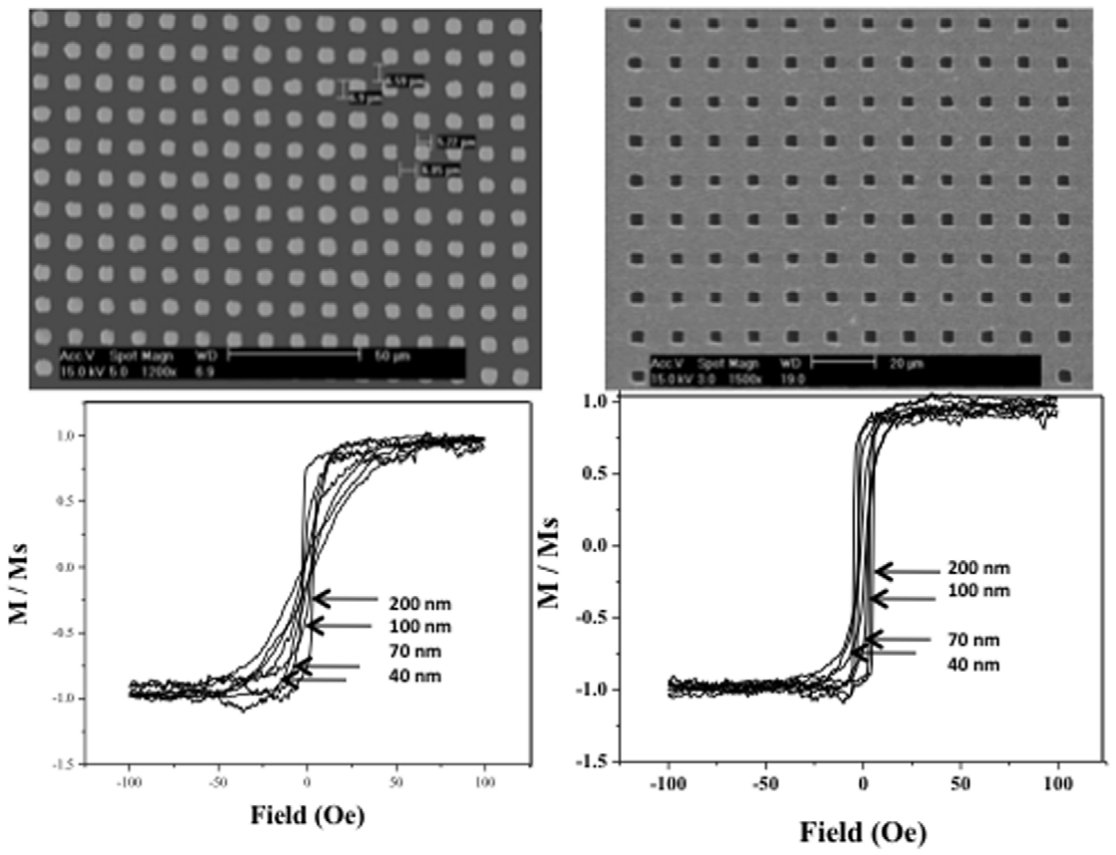
SEM image of 5 µm and 3 µm magnetic particles on a silicon surface (top, right and left) and VSM graphs (bottom, left and right) of 40, 70, 100, and 200 nm thick magnetic particles on silicon surface. The 5 and 3 micron particles were printed using nickel mesh masks with 5 and 3-micron openings. Particle size standard deviation was approximately 7% of the mean based on ImageJ analysis. The evaporation of various thicknesses of magnetic layers creates the difference in the coercive field of the particles. The coercivity of the particles increases with increasing magnetic layer thickness. The VSM measurements were taken with the easy axis of the particles’ magnetization oriented along the direction of the applied magnetic field.

Particles of various sizes and shapes were printed using AAL. Figure 2 shows SEM images of (A) 5 µm magnetic particles made using a 5 µm nickel mesh mask; (B) 3 µm magnetic particles made by narrowing down the openings in the 5 µm nickel mesh to 3 µm by sputtering copper onto the mask; and (C and D, respectively) results of VSM measurements for the 5 and 3 µm particles.

The 3- and 5-µm particles were printed using a nickel mesh mask with area of 1 cm^2^ containing 5 µm openings on a 10 µm pitch, which was used directly to fabricate the 5 µm particles, and used to make the 3 µm particles with the openings closed down to 3 µm using conformal sputter deposition of copper. The average size of the particles was 3.00 and 5.00 µm with standard deviation of 0.21 and 0.34 µm respectively, which corresponds to approximately 7% in each based on analysis using ImageJ for microscopy (version 1.41 n, Oct., 2008) [27]. The size deviation of particles may have come from the openings in the mesh itself or from variations in etch time (refer to Figure 1, step (c)).

Tri-layer sandwiches of 10 nm gold/permalloy (of thickness 40, 60, 70, 90, 100, 130, 160, 200, and 300 nm)/10 nm gold were evaporated using a thermal evaporator in the presence of a magnetic field of 40 Oe. Metals were deposited at vertical incidence, and the pressure inside the chamber was approximately 7.5×10^−6^ Torr. The evaporation steps were performed in the presence of a magnetic field to enhance the uniformity of the magnetic properties of the material; VSM measurements of particles fabricated in the absence of a magnetic field showed higher noise levels.

**Figure 3:**
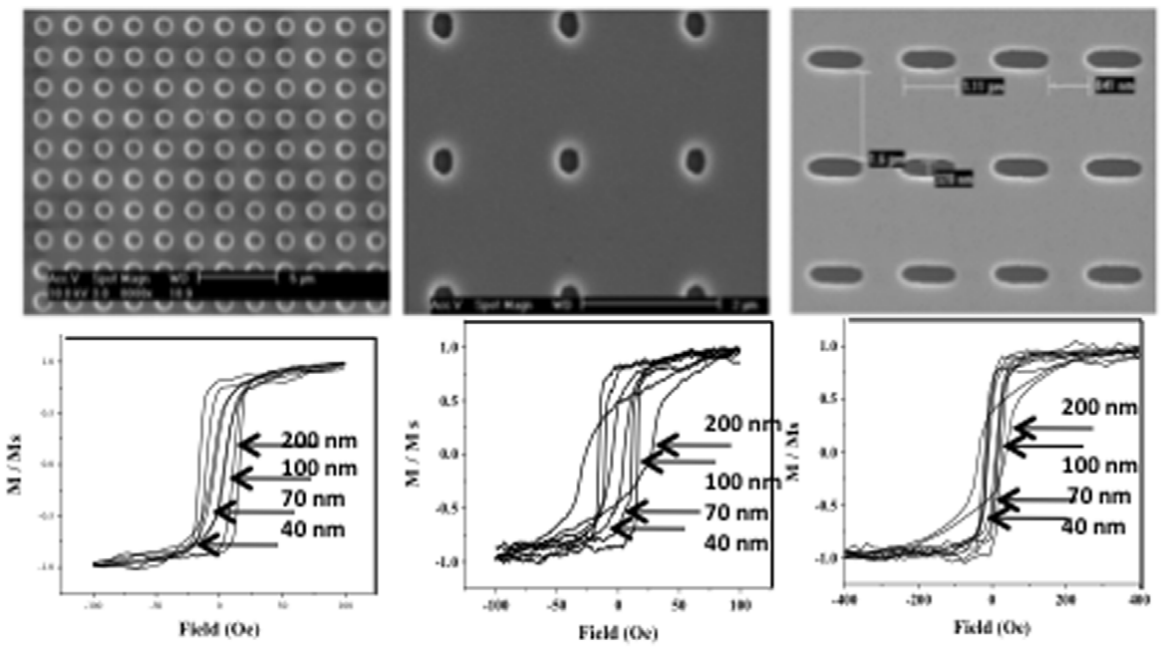
SEM image of 1micron (A), 300 nm (B), rod-shaped particles (C) and their corresponding VSM measurements (D, E, and F). The shape anisotropy dominates the material properties of the 300 nm thick evaporated particles, resulting in higher coercivity and lower remnant magnetization. The results of VSM measurements of rod-shaped particles show the highest coercivity among all particles, measuring as high as 37 Oe and remnant magnetization decrease from 0.7 to 0.3 with increase of layer thickness.

To make the 1 micron, 300-nm, and rod-shaped particles (Figure 3), a silicon nitride stencil mask with 300 nm circular openings was used. The average size of the silicon nitride hard mask openings by ImageJ analysis of SEM images was 300±17.3 nm (mean±SD), corresponding to a 5.8% size deviation among the mask openings.

### Magnetic Property Dependence on Permalloy Thickness

The coercivity curves (loops of normalized magnetization, M/M_S_, as a function of applied magnetic field, H loops) of various-sized particles were measured along the easy axis for permalloy thicknesses of 40, 60, 70, 90, 100, 130, 160, 200, and 300 nm, while keeping the top and bottom gold layers at 10 nm each. As shown in Table 1, the coercivity does not vary much for thicker layers of permalloy for 5 and 3 µm particles due to their approximation to a continuous film, so the shape anisotropy does not exhibit itself at this point. As the thickness of the permalloy layer increases, the remnant magnetization, M_R_, of the 5 µm particles decreases, suppressing aggregation and promoting particle stability in liquid suspension.

**Table 1:** Summary of coercivity and remnant magnetization of magnetic particles with permalloy layers 40–300 nm thick.

Particle Size	Coercivity (Oe)	Remnant Magnetization
Rods	10–15	0.3–0.7
300 nm	6–8	0.7–0.9
1 µm	3–8	0.3–0.85
3 µm	2–5	0.3–0.85
5 µm	3–5	0.07–0.75

The magnetic properties of the 3 µm particles are similar to those of the 5 µm particles. The coercivity remains within 2 to 5 Oe for a given range of magnetic layer thicknesses. The remnant magnetization is slightly higher for 3 µm than for 5 µm particles, decreasing from 0.85 to 0.3 as the thickness of the permalloy layer increases.

As can be seen from Figure 3D, 1 µm particles exhibit different magnetic behavior than the 5 and 3 µm particles due to their size and geometry. The remnant magnetization decreases from 0.85 to 0.4 as the thickness of the permalloy layer increases from 40 to 200 nm. The coercivity increases with increasing permalloy thickness, from 3.5 to 18 Oe. Interestingly, for 300 nm thick particles, as the permalloy layer becomes thick enough to influence the geometry of the particles, contributing more to the shape anisotropy, the M-H loop takes a different shape, exhibiting higher coercivity, lower remnant magnetization, and a wider distribution of the switching field.

For 300 nm diameter particles, Fig. 3E, the resulting H-M curves exhibit remnant magnetization from 0.6 to 0.9, while coercivity changes from 6 to 9 Oe for permalloy thicknesses from 40 to 200 nm. At the 300 nm thickness, when the shape anisotropy of the particles begins to dominate the material properties, the M-H loop shows a wider distribution of switching field. The coercivity of those 300×300 nm cylindrical particles increases up to 25 Oe.

### Coercivity and Remnant Magnetization of Magnetic Particles

**Figure 4:**
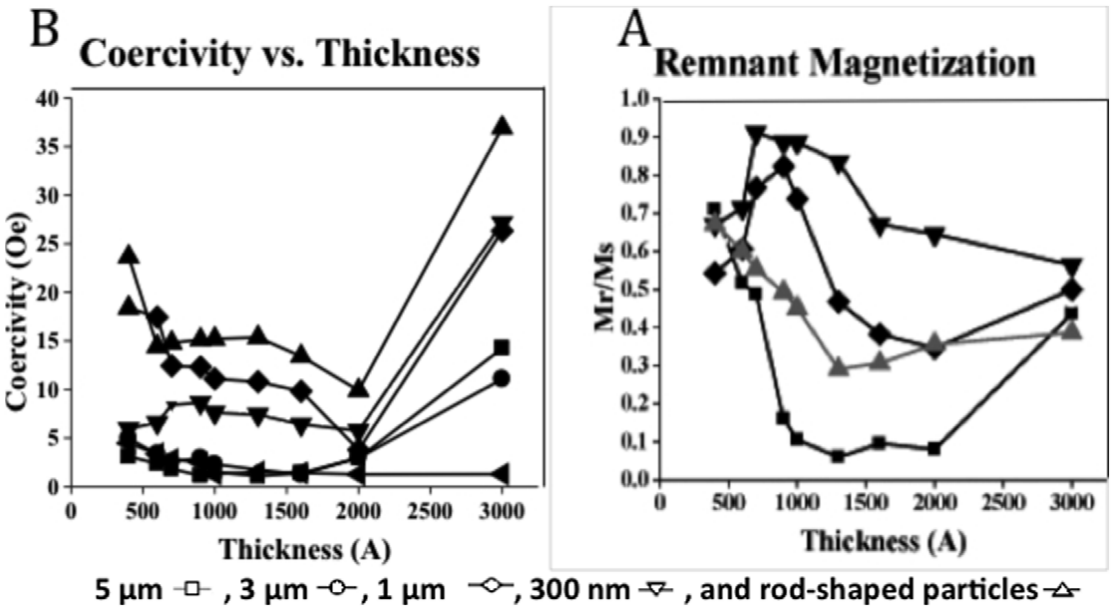
Coercivity curves for 5 µm, 3 µm, 1 µm, 300 nm, and rod-shaped particles. The coercivity (A) and the remnant magnetization (B) values were extracted from the VSM plots described above for different particle sizes and varying magnetic layer thicknesses.

Figure 4A shows the coercivity curves of the magnetic particles made with various permalloy layer thicknesses as described above. The samples were placed vertically in the VSM and the magnetic field was applied parallel to the surface of the samples. The M-H loop for each sample was measured 3 times and the average M-H curve was plotted. The coercivity for each sample was obtained by calculating the magnetic field at zero magnetization. The remnant magnetization extracted from the M-H loops was lowest for the 5 micron structures (Figure 4B) in the range of 100 to 200 nm permalloy thickness. It is important that the particles of interest have the lowest remnant magnetization possible, in order not to aggregate in solution.

### Rod-shaped Particles

To make rod-shaped particles, multiple exposures of the stencil mask were printed as the substrate was moved horizontally. The resulting particles have dimensions of 1.0×0.3 µm, as shown in Figure 3C. As shown in Figure 3F, a higher magnetic field was required to saturate the magnetic rods, compared to other-shaped particles of similar permalloy layer thickness. The rod-shaped particles show high remnant magnetization and a coercivity of 10 to 15 Oe. The major change can be noticed starting at a permalloy thickness of 300 nm, where the dimensions of the particles (the ratio of thickness:width:length) changes to promote the shape anisotropy.

**Figure 5:**
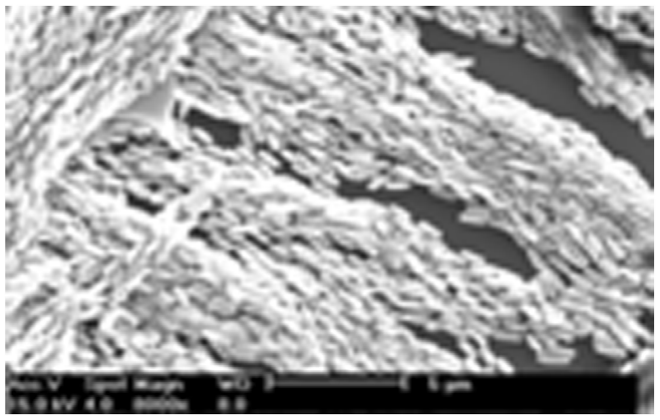
SEM image of preferred orientation of 400 nm thick rod-shaped magnetic particles. The particles were fabricated using horizontal movement of the AAL stage and evaporation of gold/permalloy/gold tri-layers. The sample was lifted-off in TMAH solution, rinsed in DI water to remove the traces of TMAH and dried on a silicon surface in the absence of any external magnetic field. The observed preferred orientation of the particles is the result of shape anisotropy dominating the material properties. The rods’ alignment into the long-stranded pattern is the manifestation of the switching of easy axis along the length of the particles due to their shape.

Among all the particles made, only the 400-nm thick rod-shaped particles showed a preferred magnetic orientation, as shown in Figure 5. After rinsing with DI water, the particles were dried on a bare Si substrate. Even in the absence of an external magnetic field, long chains of rod-shaped particles were formed by self-orientation.

### Anisotropic Magnetic Properties of Rod-Shaped and 300 nm Magnetic Particles

**Figure 6:**
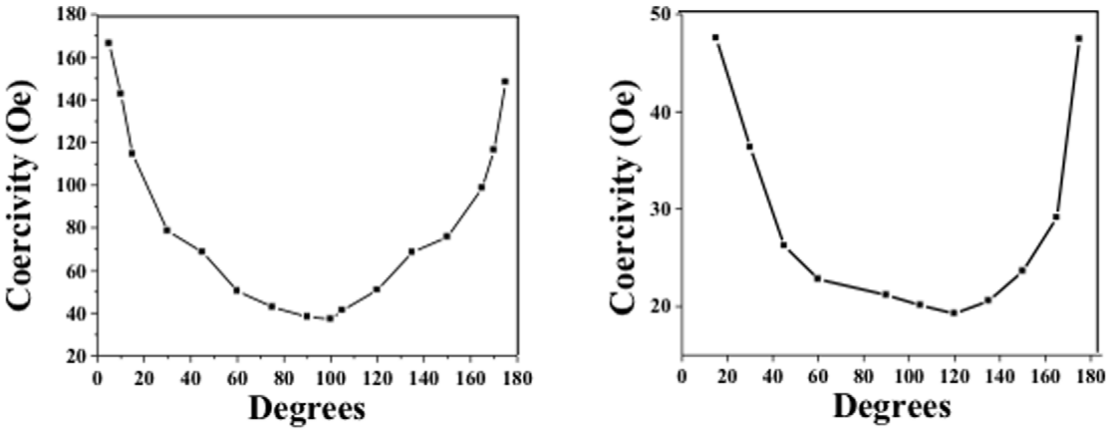
Rotation of rod-shaped particles (A) and cylinder-shaped particles (B) along the easy axis. The particles were printed to achieve specific geometries, which allows the manifestation of shape anisotropy. The samples were placed in the VSM with the easy axis parallel to the direction of the applied field and rotated in increments of 15 degrees to measure the change in coercivity as the sample moves from easy axis to hard and further to easy axis, making half-a-turn of 180 degrees. Cylindrical particles 300 nm in diameter and 400 nm tall exhibit a coercivity of approximately 47 Oe, while the rod-shaped particles 300 nm wide, 400 nm tall and 1.2 micron long exhibit a coercivity of 167 Oe.

The magnetic properties of the particles were examined while rotating the sample 180° along the easy axis of magnetization in VSM to determine the switching field. For most particles, because of their size and shape, it was found that the difference in magnetization along hard and easy axis was negligible. However, for the circular and rod-shaped gold/permalloy/gold particles with 300 and 400 nm-thick permalloy, a difference in magnetization was observed. Figure 6A, shows the coercivity measured for cylinder-shaped particles 400 nm in height and 300 nm in diameter by rotating the sample from the easy axis to hard axis and back to easy axis in increments of 15 degrees. The resulting geometrical shape and dimensions contribute to the switching of magnetic field while rotating the sample perpendicular to the height (the easy axis) of the cylinder. The highest coercivity was measured to be 47 Oe along the easy axis.

The gold/permalloy/gold layers were evaporated to form rod-shaped particles 1.2 µm in length, 300 nm in width, and 400 nm in height. The VSM measurements were performed at every 15° from 5° to 175° and the highest coercivity obtained was 167 Oe along the easy axis of the sample. Figure 6B shows the coercivity measured from the rotation experiment. In this scenario, the easy axis of the rod-shaped particles is at 90 degrees when compared to the easy axis of the previously explained cylindrical particles. The magnetization induced during the formation of the particles due to the presence of magnetic field, as for all the other particles, is along the top surface, which has an area of 3.6*10^5^ nm^2^. When comparing this number to the top surface of the cylindrical particle, which is 2.8*10^5^ nm^2^ one can see that the magnetized surface of the rod-shaped particles is larger than the surface of the cylindrical particles.

### Conclusions

In this work, we have demonstrated the ability of ion beam aperture array lithography to fabricate a range of particles of high uniformity, and flexible shape and composition, with vastly higher throughput than electron-beam lithography. The AAL was used to fabricate a range of gold particles with permalloy cores of varied thickness, and their magnetic properties were characterized by VSM.

The highest coercivity was observed for rod-shaped magnetic particles and the lowest remnant magnetization was measured for 5 µm square magnetic particles. Both the coercivity and remnant magnetization of the particles decreased with increasing particle size. Since for biological applications it is more favorable that the particles will not agglomerate in solution, the remnant magnetization of the particles should be minimized.

In future work, the 5 µm particles can be subjected to surface functionalization by self-assembled monolayer chemistry approach. Future work also will further enhance the uniformity of the particles by using a mask with more uniform openings.
